# De-stabilizing innate immunity in COVID-19: effects of its own positive feedback and erratic viraemia on the alternative pathway of complement

**DOI:** 10.1098/rsos.221597

**Published:** 2024-01-17

**Authors:** Jonathan Reeve

**Affiliations:** Senior Research Fellow, Nuffield Department of Orthopaedics, Rheumatological and Musculoskeletal Sciences, University of Oxford Botnar Research Centre, Windmill Road, Oxford OX3 7LD, UK

**Keywords:** innate immunity, SARS-CoV2 infection, *in silico* modelling, viral reproduction dynamics, alternative pathway of complement, positive feedback

## Abstract

Complement provides powerful, fast responses in the human circulation to SARS-CoV-2 (COVID-19 virus) infection of the lower respiratory tract. COVID-19 effects were investigated in a revised human *in silico* Mass Action model of complement's alternative pathway (AP) responses. Bursts of newly circulating virions increased the fission of Complement protein C3 into C3a and C3b via stimulation of the lectin pathway or inhibited complement factor H. Viral reproduction sub-models incorporated smoothly exponential or step-wise exponential growth. Starting complement protein concentrations were drawn randomly from published normal male or female ranges and each infection model run for 10 days. C3 and factor B (FB) syntheses driven by Lectin Pathway stimulation led to declining plasma C3 and increasing FB concentrations. The C3-convertase concentration, a driver of viral elimination, could match viral growth over three orders of magnitude but near-complete exhaustion of circulating C3 was more prevalent with step-wise than with ‘smooth’ increases in viral stimulation. C3 exhaustion could be prolonged. Type 2 Diabetes and hypertension led to greatly increased peak C3-convertase concentrations, as did short-term variability of COVID-19 viraemia, pulmonary capillary clotting and secondary acidosis. Positive feedback in the AP greatly extends its response range at the expense of stability.

## 1. Introduction

Complement proteins are expressed within immune T-cells and in plasma [1]; circulating complement is especially interesting being the part of Innate Immunity with dynamics that can be analysed quantitatively with the law of Mass Action [2]. COVID-19 might lead to its excessive plasma activation through the lectin pathway [3–8] or as in severe Dengue virus infection [9] through reducing complement factor H (FH) activity [10]. This focuses attention on complement's alternative pathway (AP) whose regulation has been recently reviewed [11]. Many gaps in our knowledge remain concerning the reproduction and release from infected cells of the COVID-19 family of viruses, requiring further investigation [12]. Of particular interest is its use of lysozymes for egress [13] and the day-to-day (or more rapid) variability in virus concentrations in the infected [14]. The complexity of innate immunity suggests a need for dynamic modelling. But so far, published modelling studies of the innate immune system have only represented a single subject [15,16] or contrasted a typical normal subject with a subject with a rare disease of interest.

An updated Mass Action analysis of the AP—excluding effects downstream of the C3-convertase—is proposed for investigating the effects of healthy human variations in upstream complement protein concentrations regulating circulating C3-convertase. This analysis then scopes the more likely effects of pulmonary COVID-19 infections that stimulate the lectin pathway [17] or inhibit the protective complement protein Factor H (FH) [18]. Through its effects on MBL-associated serine proteases (MASP) synthesis, this coronavirus is associated with increased C3a and C3b generation from circulating C3 protein, while FH inhibition would further increase the availability of C3b for synthesis of the C3- and C5- convertases. While information on variations in the statistical distributions and time-courses of pulmonary viral COVID-19 proliferation from infected type 2 pneumocytes is incomplete, effects on innate immunity of these infections cannot be fully understood. This still permits the study of the likely responses of the AP to varied amplitudes and time-courses of COVID-19 viraemia in the 10 days before an acquired immune response develops.

Here I examine in simulation the effects of natural variations in different simulated hosts' innate immune systems on his or her predicted response to several proto-typical pulmonary COVID-19 infections. Some were associated with partial clotting of the pulmonary capillaries insufficient to cause death by asphyxia and were aligned with recently published data. While the results revealed fairly predictable responses of innate immunity when the circulating viral load increased along a smoothly enlarging growth curve, discontinuities in viral release from infected pneumocytes, leading for example to sudden orders of magnitude increases in viral particle concentrations, were found capable of disabling the Pathway through exhaustion of complement C3. This suggests that innate immunity, like many regulatory systems incorporating positive feedback, might become functionally disabled by one or more specific stimuli, in this case viral.

## 2. Methods

### 2.1. Models of COVID-19 virus infection

To be consistent with Bar-On *et al*.'s review [19], it was assumed that the latent period (cellular level) defining the time from the infection of a type 2 pneumocyte to release into pulmonary capillaries of functional daughter extracellular virions was 48 h. Although the burst size of a single pneumocyte infected with COVID-19 might be 10^4^ virions, it was also assumed that the number of infectious units released would be smaller by approximately a factor of 10^3^ as argued by Sender *et al*. [20], so that the growth in the lectin pathway MASP stimulus to the fission of C3 into C3a and C3b over each 48 h period would grow exponentially in the region of 10-fold. Since the number of type 2 pneumocytes are thought to greatly exceed the numbers initially infected by a factor of about 10^3^ [20], and due to the high local density of ACE2 receptors, it was also assumed that released infectious units were, due to their high affinity for the receptor, highly likely to infect adjacent previously uninfected pneumocytes before they could leave the pulmonary capillary bed and enter the systemic circulation.

**Figure 1.**
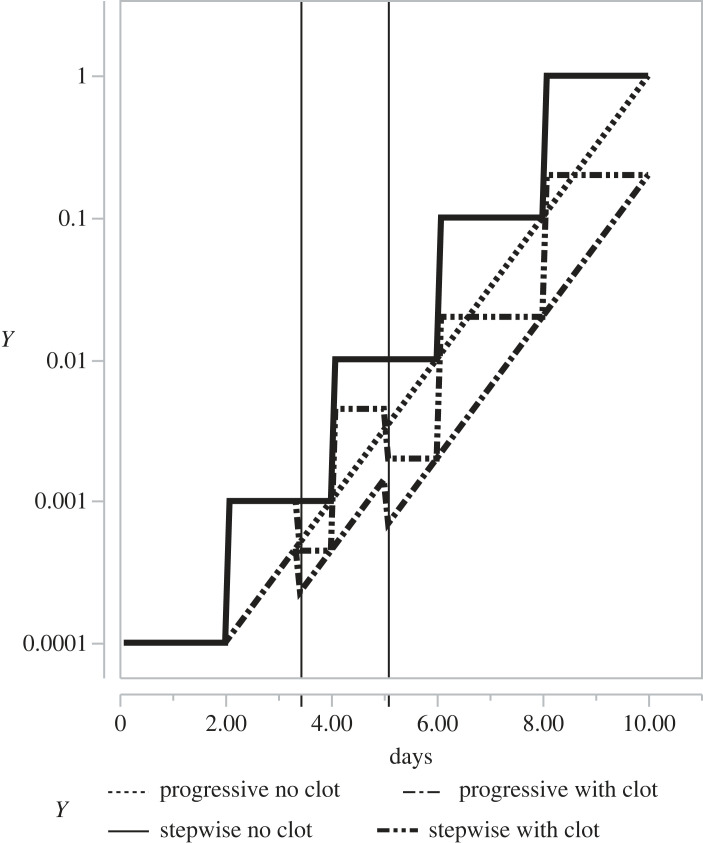
‘Tickover’ plus MASP stimulation inputs driving C3 synthesis in various versions of the model. Up to 2 days, in all simulations, the only input stimulating C3 synthesis was tickover (0.0001 arbitrary units ordinal scale), which matched the combined effects of C3 destruction due to normal degradation plus the combined effects of FH and FI that removed from the circulation free C3b generated from C3 by the effects of the C3-convertase driven positive feedback loop (shown in figure 2 with rate constant *k*_3_). Between 2 days and 10 days, there was an increasing viral stimulus that grew exponentially at the rate of 10-fold every 48 h.

Although released from infected cells by budding [21], the time-course of COVID-19 release from infected cells over the 48 h since infection remains unclear. Therefore, two contrasting patterns of viral release into the extracellular fluid were modelled: a sudden step-wise 10-fold increase every 48 h; and a smoothly developing exponential increase growing at the equivalent rate of 10-fold over 48 h. In all simulations, it was assumed that half of the 4000 lung lobules [22] were infected initially and in some simulations also that the capillary blood supply to infected lobules was partially interrupted at an arbitrary 3.42 days by sudden complete clotting affecting 55% of infected lobules. In simulations affected by clotting, a second episode of clotting was programmed at 5.08 days leaving just 20% of infected lobules with viral access to the general circulation [23]. The resulting four patterns of Lectin Pathway stimulation tested in the simulated AP are shown in figure 1.

### 2.2. Modelling framework and scope

The AP was evaluated using the updated model over a sequence of short 120 min periods during each of which the Steady State was maintained transiently. All four known feedback activities included in the model changed as one 120 min period transitioned to the next. Feedback was thereby implemented after a fixed 120 min time delay. All regulatory reactions not involving feedback were assumed to be instantaneous. Negative feedback regulation included both intrinsic negative feedback arising through consumption of C3 and complement factor B (FB) [24]; while positive feedback, as well as the long appreciated effect of the C3-Convertase to increase formation of C3a and C3b from C3, was supplemented by the effects of C3a to indirectly increase C3 and FB substrate synthesis in the liver and elsewhere [4]. The combined effects of factors H (FH) and I (FI) to destroy unattached C3b provided negative feedback.

### 2.3. Updated model equations

In these equations, complexes of two or more complement proteins are indicated as follows: a complex of FY with FZ is shown as FYFZ. The equation governing the C3-convertase concentration was shown previously [24] to be a rectangular hyperbola, differing from a Michaelis–Menten equation (also a rectangular hyperbola) in having a negative rather than a positive constant *β*:

2.1 $$\frac{1}{Y}=\left(\frac{\alpha }{X}\right)-\beta ,$$

where *ϒ* is the C3-convertase concentration, *X* is a complex function of several regulating reaction rate constants and precursor concentrations that crucially includes the ratio of the rate of splitting of C3 into C3a and C3b (plus any ordinary degradation) to the functional FH concentration, while *α* and *β* depend on a subset of rate constants and *β* is proportional to the rate of ‘tickover’ [25] supplemented by any additional AP activation, through the activity of the lectin pathway. The original analysis involved solving four algebraic Mass Action equations in four unknowns—these being the plasma concentrations of C3, C3b, C3bFH and a quantitative function of the various C3b-factor B complexes that included those with circulating properdin, collectively termed C3bBt [24]. This model was revised to accommodate the relationship of properdin with C3b and the regulation of the dissociation of the C3-convertase complex that occurs through interaction with FH directly into C3b, Bb and FH, estimated by Bakshi *et al.* [16]. The four original simultaneous equations then became (with the symbol P representing properdin; *f*_1_ the ratio of functional C3-convertase complexes to total C3B complexes; and *f*_2_ the ratio of properdin unbound to total C3bB complexes). The numerators on the right side of these four equations represent formation rates and the denominators fractional removal rates.

2.2 $$[C3]\;=\frac{K}{\left(\;f_{1}k_{1}[C3b.\mathrm{FB}t]+\Lambda \right)},$$

(included within *Λ* is the sum of activation by the lectin pathway, activation by the classical pathway, in this paper set at zero; and in addition the contribution of C3 tickover).

2.3 $$[C3b]=\frac{(f_{1}k_{1}[C3][C3\mathrm{bFB}t]+\Lambda [C3]+f_{2}k_{3}[C3\mathrm{bFBt}]+k_{4}[C3\mathrm{bFH}])/\left(k_{5}[\mathrm{FB}]+k_{6}[\mathrm{FH}])+k_{10}[C3b.P\right]}{k_{5}[\mathrm{FB}]+k_{6}[\mathrm{FH}]+k_{9}[P],}$$

(where [C3b.*P*] = *k*_9_[*P*][C3*b*]/*k*_10_)

2.4 $$\frac{\left[C3bBt]=k_{5}[\mathrm{FB}][C3b\right]}{k_{1}f_{2}}$$

and

2.5 $$[C3\mathrm{bFH}]=\frac{k_{6}[\mathrm{FH}][C3b]}{\left(k_{4}+k_{7}[I]\right)}.$$

Published reaction rate determinants (indicated by *K*, *Λ*, *k*_1–10_) are described in table 1.

**Table 1.** Mean concentrations of male complement proteins in health.

analyte concentrations, μM, in health				s.d., μM reference table 1 in Bakshi *et al.* [16]	
C3	7.0			1.5	
FB	2.2			0.44	
FD	0.08			0.03	
FH	2.5			0.63	
FI	0.40			0.056	
properdin	0.39			0.12	
rate determinants with sources – all numbering of suffixes from Reeve & Woo and figure 2					
**name**	**value**	—	**source reference**	**units**	**coeff. of variation**
*k*_2_	107		[19]	min^−1^	
*k*_1_	8.32^a^	{*k*_2_/(5.86 + [C3])}	[26]	μM^−1^ .min^−1^	5%
*k*_3_	0.46		[26]	min^−^	5%
*k*_4_	195		[27]	min^−1^	5%
*k*_5_	0.82		[28]	μM^−1^ .min^−1^	pH sensitive
*k*_6_	312		[27]	μM^−1^ .min^−1^	pH sensitive
*k*_7_	78		[27]	min^−1^	5%
*k*_8_	130		[29]	min^−1^	5%
*k*_9_	0.87		[30]	μM^−1^ .min^−1^	pH sensitive
*k*_10_	0.028		[31]	min^−1^	5%
*k*_11_	0.82				5%

^a^when [C3] = 7.0 µM.

This update was assumed not to alter the equations for the ratio of *f*_1_ : *f*_2_ (previously calculated as {(1 + *k*_9 ·_ [P]/*k*_10_)/(1 + *k*_3_/(*k*_8 ·_ [FD])}—see appendix). The hyperbolic nature of the relationship between C3-convertase activity and a function of the concentrations of FH, FD, FB, C3, properdin and FI that determined C3-convertase concentrations consequently remained of a similar algebraic form, now accounting for the dual effect of FH to react with the C3-convertase as well as with C3b to reduce both concentrations.

Solving these equations, a rectangular hyperbola is obtained describing the relationship between C3-convertase activity and its determinants, which is of the same general form as eqn 6 in Reeve & Woo [24] *viz*:

2.6 $$\frac{1}{Y}=\left(\frac{\alpha }{X}\right)-\beta ,$$

where *α* = *k*_3_*k*_6_/(*Λ ·* k_5_) and *β* = *k*_1_/*Λ*, the quantitation of *X* being revised to:

2.7 $$\begin{array}{r} X=\frac{\left[\mathrm{FB}][C3\right]}{\left[\mathrm{FH}\right]}\;\cdot \;\frac{\left(1+k_{9}[P]/k_{10}\right)}{\left(1+k_{3}/\left(k_{8}\cdot [\mathrm{FD}]\right)\right)}\;\cdot \;\frac{k_{5}(k_{4}+k_{7}[\mathrm{FI}])}{k_{5}k_{7}[\mathrm{FI}]-0.055k_{9}[P](k_{4}+k_{7}[\mathrm{FI}]).} \end{array}$$

the portion of the equation on the right that allows for the buffering of C3b by P and shown in ***bold italics*** now replaces the term (1 + *k*_4_/*k*_7_[I]), the factor 0.055 being calculated from the results of Bakshi *et al.* [16].

The two forms of the complex of C3b with FB, open and closed, were, with algebraic advantage, combined without creating bias, so that the total pool of C3bFB was calculated to be 1.324 times the size of the open form [16]. To maintain the output of C3bFBb, under the influence of FD, at its correct rate as calculated by Bakshi *et al.*, rate constant *k*_8_ was proportionately reduced and it was further assumed that this reaction could be represented as first order as previously argued [16]. It then remained possible to solve four simultaneous Mass Action equations (numbered 2–5) in four unknowns. This was repeated for each successive 120 min period under study.

### 2.4. Modelling strategy

**Figure 2.**
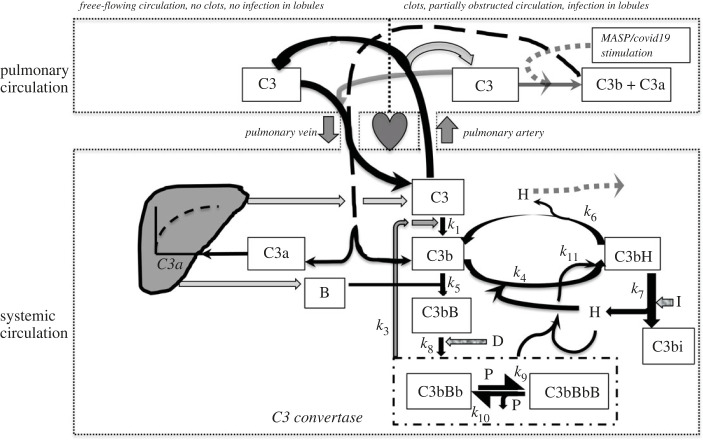
Block diagram of the main components of the model (updated from the block diagram of Reeve & Woo [16]) now located in the two circulations, pulmonary and systemic. Note that the usually prefixed ‘F’ as in FB for factor B is omitted in this diagram and should be assumed. P stands for properdin and rate constants are indicated by lower case *k*_1–11_. Translocations and transformative actions are indicated by arrows and dashed arrows indicate events associated with the stimulation of the lectin pathway in some circumstances (increased C3a and C3b formation and possibly disablement of FH). In the systemic circulation are located the principal components of the AP, including metabolic transformations and feedback loops (like circulatory flows shown by arrows). C3 and FB synthesis are assumed in the model to occur in the liver. In the absence of infection, C3 flowing through pulmonary lobules on the left is returned unchanged to the systemic circulation. In the presence of lobular infection, C3 may either be returned to the systemic circulation unchanged or after being split into C3a and C3b. Clotting is assumed to reduce flow through the infected part of the pulmonary circulation. Factor D acts as a catalyst. Some proteins have never been found (e.g. C3bH, representing C3bFH): but their presence here helps illustrate that the three way interaction between C3b, FH and FI might not be absolutely instantaneous. The Michaelis–Menten equations regulating C3 and FB synthesis with their maxima are modelled as though functioning within the liver's ‘parenchyma’, being of the forms: for C3 synthesis: 15*C3a formed/minute in previous 120 min *pre-infection C3a formed/minute/(C3a formed/minute in previous 120 min + 14*pre-infection C3a formed/minute) and for FB synthesis; 40*C3a formed/minute in previous 120 min *pre-infection C3a formed/minute/(C3a formed/minute in previous 120 min + 39*pre-infection C3a formed/minute) [32].

A ‘healthy’ model was classed as being devoid of any extraneous stimulus to AP activation such as infection; in consequence, the lectin and classical pathways were assumed inactive in health and tickover metabolism of C3 [25] continued with basal replacement of any C3 or FB lost through non-activation mechanisms supporting the generation of new convertase molecules. In modelling the Convertase-stimulating effects of a COVID-19 infection, the tickover metabolism of C3 was supplemented in proportion to the level of COVID-19 infection in an individual lung lobule. The overall effect of an infection was then calculated as the sum of these effects in individual lobules. In some simulated infections, FH was also inhibited. The general structure of the model is shown in figure 2. Positive feedback, resulting from the effect of C3a to indirectly promote C3 and FB synthesis [32] as well as the effect of FH to promote the inactivation of C3b attached to FBb additionally to its effect on free C3b [16], are key differences from the old Reeve and Woo model. It was assumed for simplicity that in each sex C3, FB and other complement proteins typically have volumes of distribution in the well-mixed extracellular fluid equal to 1.4-fold the assumed plasma volume of 3 l [33] and also that resting cardiac output delivers 2.75 l of plasma to the pulmonary and systemic circulations per minute.

### 2.5. Model implementation

Data in all models were expressed in the following dimensions: time minutes; volumes litres; mass Moles. The normal data set used to populate the pre-infection model of normal AP function was similar to that recently assembled from the literature by Bakshi *et al*. [16], with other published data used as indicated previously. Because a key aim was to study effects of biological variation across a population of infected adults, a statistical program, JMP 13 (which generated random series useful for mimicking reported statistical distributions) was used to implement relationships between the various proteins comprising the AP as regulated by the law of Mass Action. Because in real time biological feedback often gives rise to differential equations that could not be implemented on this platform, the feedback processes in the model required the approximate solutions referred to previously. In this paper, C3 and FB concentrations were updated every 120 min to allow for the stimulating effects of C3a on their syntheses (figure 2) and the above simultaneous equations (2.2)–(2.7)) re-solved. Proteins not consumed in the processes of generating the C3-convertase were maintained at constant concentrations (FD, FH, FI and properdin), except when the effects of simulated viral reductions in functional FH were tested.

#### 2.5.1. Biologically plausible variations in regulatory variables in health

Biological variations between different individuals in complement protein concentrations were programmed to follow normal distributions in the absence of contrary evidence. Graphically published normal data were mostly suggestive of statistical normality [34–36]. One exception was male FD concentrations that appeared to be better fitted by a lognormal distribution [36]. The means and published or calculated standard deviations used are listed for individual proteins in table 1. Arterial pH may be disturbed by COVID-19 lung infection, reportedly in the range 7.0–7.4 in fatal cases [37]. Three rate constants regulating the AP are influenced by pH [38].

#### 2.5.2. ‘Monte Carlo’ methodology for investigating the effects of host biological variation

Men and women were studied separately because of sex-related differences in some AP protein concentrations, with sex-specific versions of the model solved repeatedly. The initial dataset, representing the reported medians of the distributions of complement protein concentrations and their regulatory rate and Michaelis constants in healthy individuals, was modified in the first and subsequent iterations to include appropriate randomly generated deviations in initial conditions and rate constants. The initial model was thereby used to generate a set of models representing a randomly selected group of men or women experiencing *in silico* an identical pattern of COVID infection.

While the coefficients of variation for complement protein concentrations used were derived from published studies (table 1), data on the between-individual variability of the rate constants used in the model were generally unavailable. To explore the possible effects of such variability, two contrasting assumptions were made: each rate and Michaelis constant had an arbitrary coefficient of variation of either 5% or of 20%. Random variations in each analyte, rate and Michaelis constant were assumed independent of each other in the absence of evidence to the contrary [34].

To study the effects of chronically increased synthesis of C3, FB and FD associated with non-infectious disorders such as obesity, type 2 diabetes mellitus and hypertension, in some simulations the healthy male model was altered so that synthetic rates of each was increased by a factor of 1.59, other initial conditions remaining unchanged. A set of new simulations gave results that were then compared with the results derived from the ‘healthy’ male model.

#### 2.5.3. Statistical analysis of results

Because reportedly men suffered more acutely from COVID-19, initially male data were used. In between-group comparisons, 20 simulations were generated per group, with randomization of each starting value (in the case of variable analytes) or rate/Michaelis constants being generated independently. Except where otherwise stated, each starting value/rate constant was drawn from a normal distribution with its mean and coefficient of variation matched to reported normal data.

In the absence of substrate exhaustion the simulation was stopped at 10 days, because acquired immunity was expected to then start modifying the results unpredictably. In within-group comparisons, the normality of data was assessed visually with normal quantile (QQ) plots. Distributions within outcome groups were assessed for normality by the Shapiro–Wilk *W*-test and for log-normality by the Kolmogorov *D*-test. Inter-group comparisons (e.g. of the same analyte measured at the same time point) were made using quantiles and the Wilcoxon Rank-Sum test.

Backwards step-wise multivariate regression was used to examine the effects of sex and duration of infection on outcomes of interest (or their logarithms when logarithmically distributed) such as C3-convertase concentrations.

The early prediction of outcomes being clinically important, the predictability of modelled C3-convertase and other analyte concentrations was examined after two or more cycles of COVID-19 reproduction by measurable concentrations of simulated complement proteins previously input at baseline.

Decline in circulating C3 concentrations to apparent zero—termed here a ‘Capsize’ of the AP—was observed in some simulations when viral stimulation increased in a step-wise fashion. The time to capsize was made the outcome variable in Cox Proportional Hazard models [39] that included determinants of potential interest such as pre-infection complement protein levels and the presence or absence of pulmonary clotting.

## 3. Results

### 3.1. Progressive, exponential and smoothly increasing lectin pathway stimulation

**Figure 3.**
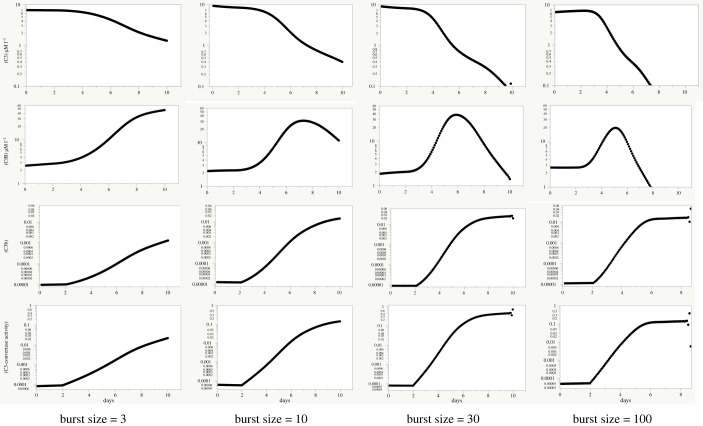
Results of range-finding to explore the effects of burst size in the progressive male model. Each column represented the same four outputs, shown on the same logarithmic scales: C3, FB, C3b and C3-convertase concentrations. In each column, burst size was varied, ranging from 3-fold per 48 h to 100-fold per 48 h. There was a highly nonlinear response to burst size, with increasing burst size leading to a more rapid inversion of the FB concentration that had marked consequences for C3b and C3-convertase concentrations. In subsequent figures, the four outputs are shown in the same order.

In preliminary range-finding, it was shown that the effective burst size [20] had large, nonlinear effects on the AP response (figure 3). This was partly attributable to the dynamic responses of C3 and FB synthesis to increased C3a synthesis, both being regulated by Michaelis–Menten equations limiting maximal synthesis rates [32]. There was, based on limited evidence [32], an approximately threefold difference in their maximal increases relative to the healthy state that favoured FB. Following Sender *et al*. [20], in the following investigations an effective burst size of 10-fold was chosen for study leading to an exponential increase in the stimulus to the AP of 10-fold per 48 h.

**Figure 4.**
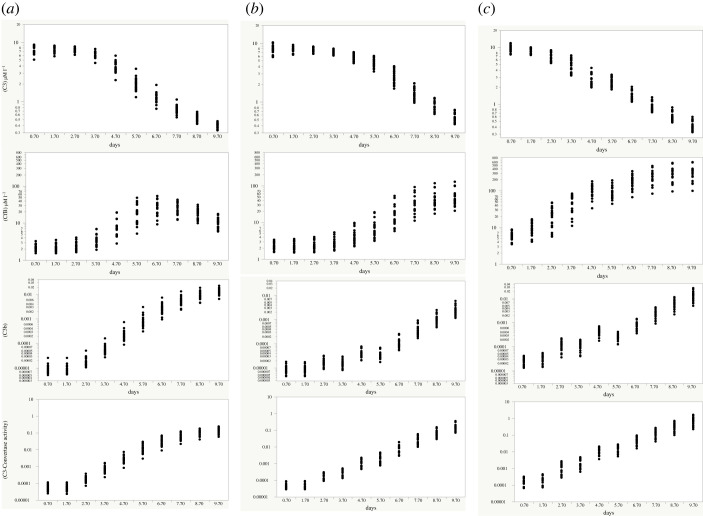
Effects of inter-individual variations in complement proteins and their regulatory rate constants. Column (*a*): the normal male model with progressive growth of viraemia at the rate of 10-fold per 48 h and in the absence of clotting. Column (*b*): the same normal male model, but with two episodes of pulmonary clotting reducing blood flow to the infected lobules by an ultimate 80%. Column (*c*): the same as Column B except that there were on average 59% increases pre-infection in both C3 and FB concentrations. Each dot represents an outcome at one point in time in a single simulation, reflecting the effects of normal inter-individual variations in complement component concentrations.

Figure 4 shows the simulated development of four key AP indices in 60 individual simulated infections in three male adult populations. In column A are shown changes in plasma [C3], [FB], [C3b] and [C3-convertase] in 20 infections in normal males unaccompanied by clotting. The time points displayed are those at which the rates of MASP stimulation of C3 fission are equal to those displayed in the results of the step-wise simulations (see following section D). In each individual infection, MASP stimulation of the fission of C3 into C3a and C3b was programmed to increase C3a formation by four orders of magnitude at the exponential rate of an order of magnitude (10-fold) increase every two days. All initial concentrations of complement proteins and regulators were drawn randomly from distributions as described in Methods. It can be seen that in these and subsequent simulations there was a tendency for [C3] to decline over time and for [FB] to rise; however [C3] was never fully exhausted in any simulation in which C3 fission increased gradually and progressively so long as FH activity remained within the normal range.

In figure 4 Column B, a further 20 simulations in normal male subjects are shown in which two episodes of pulmonary capillary clotting were assumed to occur. MASP stimulation was simulated as in Column A. Clotting that obstructed blood flow through first 45% extending to 80% of infected lung lobules delayed the progressive increase of the complement response.

To simulate the effects of disorders that increase baseline complement protein concentrations and appear to be associated with worse COVID-19 outcomes including Type 2 diabetes mellitus [40], column C shows the effects in a further 20 simulations of the same degree of clotting combined with baseline increases in [C3] and [FB] prior to infection. Baseline C3 and FB concentrations were each increased on average by 59%. MASP stimulation remained identical with the previous simulations while clotting followed the same pattern as in Column B. Syntheses of both C3 and FB were assumed capable of proportionately the same increase during infection as in healthy men (Columns A and B). Interestingly, there was now a proportionately larger fall in [C3], despite its increased synthetic rate, due to relatively higher consumption. However the other three indices increased very markedly in comparison to the simulation in which C3 and FB concentrations were normal—by 10–20 fold 5 days into the infection, this ratio falling back slightly thereafter.

To evaluate the possibility of predicting from pre- and during-infection data the likely circulating concentrations of the C3-convertase and C3b between 9 and 10 days after infection, all 60 of these simulations were entered into linear backwards step-wise regression models in which baseline [C3], [FB] and the occurrence or absence of clotting were independent variables. The C3-convertase concentration at 9–10 days was best predicted by the baseline FB concentration, with both baseline C3 concentrations and the subsequent presence or absence of clotting making no significant improvement to the model. In contrast, the concentration of C3b was best predicted by a combination of the baseline FB concentration that increased the 9–10 day C3b concentration; and the presence of clotting, which tended to reduce it.

### 3.2. The effect of sex

**Figure 5.**
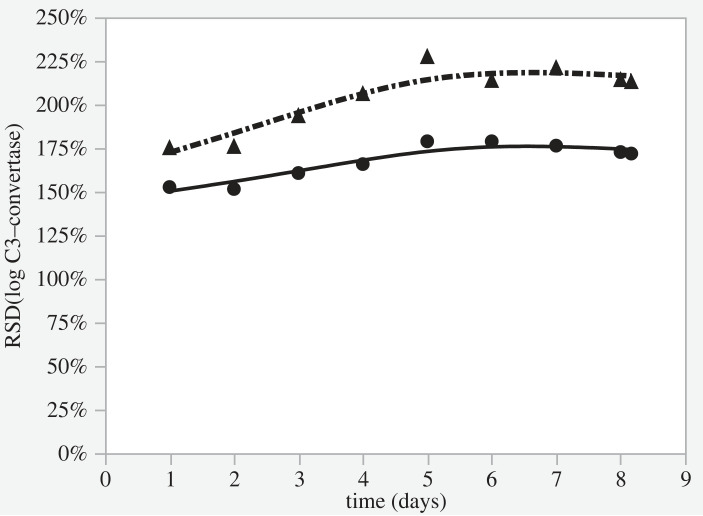
Relative Standard Deviations for the modelled C3-convertase concentration during simulated COVID-19 infections (no clotting) proceeding according to the progressive model at the rate of a 10-fold exponential increase in MASP stimulation per 48 h. A contrast is shown at the population level between males (triangles) and females (circles) in good health pre-infection. (RSD-relative standard deviation.). Data fitted by spline functions separately for men (dashed line) and women (continuous line).

After log transformation, all outcome variables in the Progressive model of normal men with COVID-19 who suffered two episodes of clotting (Column B, figure 4) were approximately normally distributed; so a further 20 simulations were generated using normal female data with the same two identical episodes of clotting. Log-transformed values of [C3], [FB]. [C3b] and [C3-convertase] were then made outcome variables in linear regression models in which time since infection and sex were independent determinants. In no case was sex a statistically significant independent determinant of any of these variables. However, the relative standard deviation across the population for the C3-convertase at all time points was significantly larger for men than for women (Fisher's *F* = 16.1, *p* < 0.0001 figure 5 and table 2).

**Table 2.** Episodes of lung capillary clotting^a^ at 3.42 and 5.08 days: modelled progression of Plasma C3 concentration in normal men and women.

day	1	2	3	4	5	6	7	8	8.2
men	mean	7.57	7.50	5.39	4.92	2.53	2.72	1.26	1.12
	I.Q.R	1.71	0.89	1.48	1.52	1.23	1.07	0.41	0.39
women	mean	7.19	7.31	5.54	5.06	2.69	2.85	1.31	1.16
	I.Q.R	1.32	0.95	1.03	1.11	1.03	0.90	0.40	0.40
P	n.s.	n.s.	n.s.	n.s.	n.s.	n.s.	n.s.	n.s.	n.s.

^a^see text. I.Q.R: interquartile range of the distribution. n.s.: *p* > 0.05.

### 3.3. Impaired factor H function

To explore the potential impact of a sustained reduction in functional FH when combined with exponentially increasing lectin pathway stimulation, four COVID-19 simulations were contrasted in which the FH concentration was drawn randomly from four male normal distributions with medians of 100%, 50%, 25% and 12.5% of the normal male median [35] and table 1. When FH was reduced by 75% or more, apparently complete disappearance of circulating C3 occurred after about the eighth day of the infection, leading to a prolonged suppression of the AP's ability to make C3-convertase. This outcome is referred to as a ‘capsize’ of the AP by analogy with the often-prolonged inversion of a racing sailboat in rough weather (in which the boat's stability is inverted through 180° of arc).

### 3.4. Lectin pathway stimulation: C3 fission increasing ‘step-wise’

**Figure 6.**
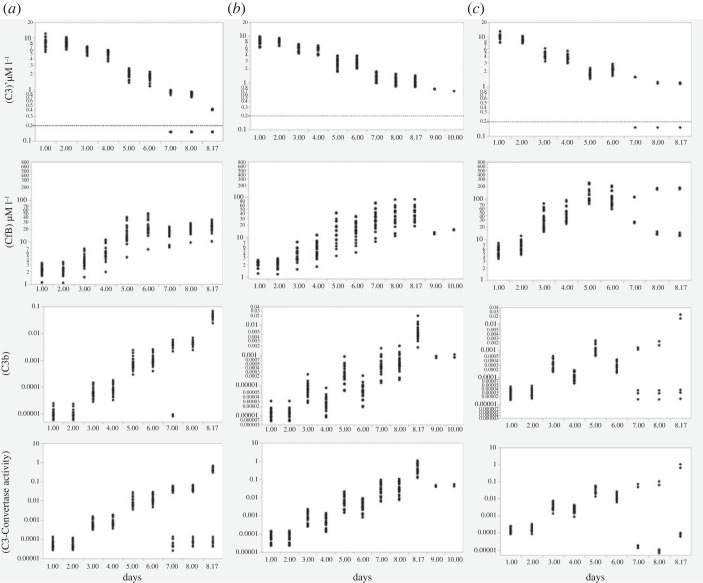
Effects of individual variations in complement proteins and their regulatory rate constants in 60 simulations with exponentially increasing, step-wise growth of viraemia at the rate of 10-fold every 2 days. Axes scaled as in figure 4. Also as in figure 4, Column (*a*) represents normal men, Column (*b*) normal men whose infection was complicated by pulmonary clotting of infected lobules and Column (*c*) men with raised levels of complement proteins pre-infection experiencing similar infections to the men in column (*b*). Comparing these results to those in figure 4, the principal differences between the effects of step-wise compared to progressive increases in MASP stimulation were the much higher risk of an AP ‘capsize’. In column (*a*), there was an approximately even split between simulated men who maintained elevated C3-Convertase concentrations beyond the eighth day, whereas in column (*b*) only two of 20 men maintained elevated C3-Convertase concentrations beyond the eighth day. In Column (*c*), just two of 20 men maintained elevated C3-convertase levels beyond the sixth day.

In this step-wise viraemia model, explored as a simple surrogate for erratically variable viremia, the viral ‘burst’ was assumed to be instantaneous and synchronized between infected pneumocytes as daughter virions are released. As above, burst size increased 10-fold with each 48 h reproductive cycle. Figure 6 shows the resulting patterns of growth or decline in C3, FB, C3b and C3-convertase concentrations. Because simulated individuals differed according to known variations in human populations, outcomes could be different. Evidently, few if any simulated ‘individuals’ could sustain a high level of AP activation throughout the 10 days. In some simulations, the potential for C3 to be metabolized immediately following a COVID-19 step-wise burst exceeded the C3 available in the circulation so that very little remained, examples of another type of AP ‘capsize’.

### 3.5. Raised baseline C3 and FB concentrations appeared to affect capsize timing

There were altogether 87 step-wise simulations, including 20 with no clotting and normal baseline C3 and FB concentrations; 40 (20 female) in which clotting ultimately obstructed capillary flow to infected lobules by 80%; 20 in which there was both clotting and raised baseline C3 and FB concentrations; and seven that were otherwise similar except that clotting obstructed flow to infected capillaries by 96%. All were entered into proportional hazard models with time to capsize as the outcome variable, while if no capsize occurred time to capsize was given a value of 10 days. The degree of clotting of infected lobules (prolonging time to capsize, *χ*^2^ = 7.4, *p* < 0.03) and raised pre-infection C3 and FB levels (reducing time to capsize, *χ*^2^ = 21.6, *p* < 0.0001) were significant independent predictors. Following a capsize, the *reciprocal* of [FB] increased empirically as a linear function of time since the start of infection, reflecting a decline in [FB] typically to 5–10 µM by the 10th day, about fivefold the normal circulating value.

## 4. Discussion

Individuals suffer very varied effects of a COVID-19 infection. A recent meta-analysis shows that several conditions associated with raised pre-infection plasma C3 levels such as diabetes mellitus are associated with worse clinical outcomes [40]; while during the progress of a COVID-19 infection another meta-analysis showed that worse clinical outcomes are associated with lower C3 levels [41]. Worse clinical outcomes therefore seem to be associated with increased complement consumption. This model provides one or more potential mechanisms for the conversion of high pre-infection C3 concentrations into low or extremely low C3 concentrations within the first 10 days of a pulmonary infection.

Because the stimulus to C3 fission created by viral infection through activation of the MASP pathway depends on the number of infected pneumocytes, it was assumed that relevant Michaelis or other constants regulating this process at the individual cell level will be unchanging, so that the change in the overall pulmonary stimulus depends on growth in the number of infected pneumocytes. During a stimulus to C3 fission into the active products C3a and C3b, the AP in this model appears capable of quantitatively matching such a viral stimulus over three orders of magnitude, which is remarkable given the tendency of some of its internal feedback to be governed by Michaelis–Menten equations limiting increases in key reaction rates to some 20–70 fold less than this maximum. This extensive response range of the AP is made possible through the actions of positive feedback by which an increasing C3-convertase concentration stimulates further fission of C3.

Positive feedback is associated with conditional instability in physical control systems; and in biology it is sometimes used to advantage e.g. in initiating parturition. In the context of complement function, evolutionary advantage might be gained by the extended dynamic range that positive feedback provides, with the proviso that instability, i.e. ‘capsizing’ or temporarily destroying the AP's function, could be either a rare adverse effect or else act beneficially as a safety valve or escape mechanism allowing a previously healthy host to evade the worst consequences of extreme levels of complement activation that can attack host cells as well as virus [10]. It is noteworthy that, in one animal model of a potentially lethal lung infection, suppressing the AP through disabling C3 synthesis was found to be protective [42].

I investigated the effects of normal, between-individual human variations in the metabolism of the circulating AP on the development of a C3-convertase response to viral stimulation through the lectin pathway, as regulated in the model through chemical Mass Action. Since in Mass Action terms, this form of stimulation can be mimicked by a proportionate inhibition of Factor H function, Lectin Pathway stimulation and factor H inhibition by COVID-19 may be indistinguishable functionally, except by separate testing. Should both effects occur simultaneously, the overall effect on the AP might at the extreme grow exponentially with an exponent raised to the power of 2 compared to biological exposure to only one effect, such that a 10-fold increase in viral stimulus delivered over 48 h could at the extreme increase AP activation by up to 100-fold.

AP instability and its causes has so far remained under-explored in studies of COVID-19 infections and appears worthy of more detailed study. During smoothly and progressively increasing lectin pathway stimulation, the present model's AP response matched the lectin pathway stimulus closely over three orders of magnitude pending the development of acquired immunity (figure 4). Moreover, as shown on the logarithmic scale, in simulated normal subjects experiencing smoothly increasing stimulation, there was no tendency for key between-individual coefficients of variation to change as the stimulus increased (figure 3*b*). Coronaviruses proliferate intra-cellularly and are then released into the circulation by budding [19]. They likely proliferate exponentially. Studies on COVID-19 release into the circulation have typically measured virus concentrations at quite long intervals rarely shorter than 24 h [14]; but the present results with the step-wise simulations suggest that release of virus in sudden ‘bursts’, should they occur over minutes or a few hours, might potentially lead to the disablement of the AP, through near-complete removal of the C3 substrate that is essential for its continued function, here referred to as a capsize.

Release of live COVID-19 virions occurs via lysosomes [13] but the dynamics of this process remain to be evaluated indicating a need for new data. If complement factor H is disabled, especially if this occurs alongside Lectin Pathway stimulation, consequently increased levels of complement activation might be followed by a failure of innate immunity as circulating C3 declines almost to zero in the 10 critical days of a typical viral infection before acquired immunity develops. For this reason, it is crucial that we understand quantitatively the processes that up-regulate C3 synthesis during challenging viral infections. The three decades old pioneering work of Katz *et al.* [32] has been insufficiently followed up.

Once circulating C3 concentrations fall near to zero, according to current knowledge the main stimuli to increased C3 synthesis disappear in parallel a short time later because of their much more rapid metabolism. Based on our current understanding, circulating C3 will then only be re-synthesized at the initially slow rate of 0.003 µM per minute [43], nearly all of which will be rapidly metabolized given the residual presence of raised FB in only slowly declining concentrations, pending the acquisition of acquired immunity.

As Box confessed, all models are wrong; but some models are useful [44]. The present model revises an old model [24] with the help of subsequently acquired quantitative data on the mechanisms regulating the AP. The current work would be useful if it directs future research towards the more important mechanisms that influence complement's role in protecting the infected subject, in the crucial days pending the acquisition of acquired immunity. Attention is drawn to the need to better understand the short-term dynamics of virus release into the circulation during the first 10 days of infection and their consequences for the complement response, both as they affect C3 and FH function. Insights are provided into how disorders such as diabetes mellitus that increase certain complement protein concentrations [38] increase the risks associated with COVID-19 as well as Dengue virus infections [9]. It is especially instructive how the functional behaviours of the various feedback pathways that help regulate the AP are amplified substantially and in ways that combine, through the existence of raised pre-infection levels of C3 and FB (as well as FD) in common chronic clinical conditions [37,45,46]. This is a consequence of a biological system working within the boundaries of Michaelis–Menten enzyme kinetics, with maxima in key reaction rates that would otherwise be unsatisfactorily low. Positive alongside negative feedback usually, in an evolutionary context, ensures stable and effective responses in the majority of *previously encountered* biological emergencies.

This work has major limitations imposed by the availability of data, which transcend the mathematical simplifications used to model the complement system. In assembling the current model, several important assumptions were necessary. The major site of circulatory C3 and FB synthesis is the liver; but it was necessary to rely on rather few data obtained in fibroblasts for numerical estimates of the Michaelis constants governing the regulation of the syntheses of these proteins [32]. As reviewed by Coulthard [47] and in line with the results of Fischer *et al.* [48], the assumption was made that synthesis of C3a completes the two positive feedback loops that regulate C3 and FB synthesis, potentially involving IL6, which are crucial in allowing an up to three orders of magnitude C3-convertase response to infection. It is not clear whether a C3a effect on circulating IL6 could, through reaching a maximum, limit further the ability of IL6 to up-regulate C3 or FB synthesis in the liver or elsewhere. New data concerning these two feedback processes most likely would require updating of the model. Because this model used difference equations to approximate differential equations, as the concentration of C3 approaches very low levels, the results become increasingly imprecise. However, despite all these limitations, what the modelling indicates is that the normal stability of the AP in health might, after certain forms of activation and for periods of days or even weeks, be ‘capsized’ in a transient stability with low or very low C3 circulating levels and high FB levels pending the clearance of the large excesses of circulating FB that can be generated in COVID-19 [42]. Finally, the involvement of the lectin pathway in COVID-19 AP stimulation is a reminder that MASPs are also intimately involved with regulating the common COVID-19 complication of blood clotting [49].

In conclusion, this investigation provides insights into how complement might usually mount a somewhat predictable response to a pulmonary COVID-19 infection within certain confidence intervals. But it also shows how the stability of complement's AP can be overcome and provides insights into how certain conditions such as raised concentrations of complement proteins pre-infection, or specific reproductive behaviour of certain viruses might disable or ‘capsize’ complement's central AP should C3 consumption then increase sufficiently to greatly reduce its concentration for days or weeks in the circulation. Two key priorities are highlighted for future investigation. First, the short-term behaviour of the COVID-19 burst and its variability needs further definition because of its likely dominating effect on the pattern of AP activation. Second, regulation of C3 and FB synthesis during viral activation of complement is shown to have a potentially profound effect on how the AP responds to infection, since this appears relevant to observational evidence that AP dysregulation is associated with clinical outcomes in COVID-19 infections [50]. Doubtless, Box's prediction that the present model like its predecessor will be proved wrong is correct. Meanwhile it has identified specific gaps in our knowledge of AP regulation, so may help accelerate the better understanding of the AP response to dangerous viral infections such as those by COVID-19 and Dengue viruses.

## Data Availability

Data available from the Dryad Digital Repository: https://doi.org/10.5061/dryad.jm63xsjhj [51].

## Appendix A. Modelling decisions regarding recently discovered complement molecules

Requiring consideration are the effects of the various C3b-containing molecules discovered since 1982. Their concentrations, whether measured by other investigators or derived indirectly, have recently been investigated by Bakshi *et al*. [16] in their appendix A1. In Bakshi's table 3 are listed the steady-state levels of these other C3bB complexes obtained using simulation of their model containing properdin, based on currently published data listed with sources in their table 1. From Bakshi *et al.*'s table 3, there are two results relevant to the present investigation. First, inclusion of properdin in their model resulted in a 58% drop in the steady-state level of plasma C3, a deviation that is unsurprising considering that the concentrations of metabolites and the reaction rate and Michaelis constants populating the model were derived from a wide variety of published sources. Secondly, among the various now-known complexes of C3b with FB and its derivatives, in Bakshi's appendix A1 that considered the steady state, C3bFBb concentrations were at least a factor of 10 larger than those of any other complex. To allow workable simplifications that seemed justifiable, in light of biological variability and other uncertainties, the effects of these non-C3bFBb complexes on the positive feedback of C3bFBb on C3 metabolism have been set at zero. This justified the continued use of the same ratio *f*_1_:*f*_2_ described in the appendix of Reeve & Woo [24], assuming that C3bFBb remains the dominant complex of C3b with FB and its derivatives [16].
